# Effect of Isovolemic, Isothermic Hemodialysis on Cerebral Perfusion and Vascular Stiffness Using Contrast Computed Tomography and Pulse Wave Velocity

**DOI:** 10.1371/journal.pone.0056396

**Published:** 2013-02-22

**Authors:** Ansgar Reising, Saskia Sambale, Frank Donnerstag, Julius J. Schmidt, Carsten Hafer, Bernhard M.W. Schmidt, Jan T. Kielstein

**Affiliations:** 1 Department of Nephrology and Hypertension, Medical School Hannover, Hannover, Germany; 2 Institute of Diagnostic and Interventional Neuroradiology, Medical School Hannover, Hannover, Germany; Harvard Medical School, United States of America

## Abstract

**Background:**

Patients undergoing hemodialysis treatment have a six-fold increased risk for stroke relative to the general population. However, the effect of hemodialysis on cerebral blood flow is poorly studied and confounding factors like blood pressure and ultrafiltration as well as temperature changes have rarely been accounted for. The aim of our study was to use state-of-the-art technology to evaluate the effect of a single dialysis session on cerebral perfusion as well as on vascular stiffness.

**Methods:**

Chronic hemodialysis patients (7 male/3 female, mean age 58 years) were recruited. Cerebral blood flow and arterial pulse wave velocity were measured before and immediately after a hemodialysis session. To exclude effects of volume changes we kept ultrafiltration to a minimum, allowing no change in body weight. Isothermic conditions were maintained by using the GENIUS single-pass batch-dialysis system with a high-flux polysulfone dialyser. Cerebral blood flow was measured by contrast-enhanced computed tomography. Pulse wave velocity was measured using the SphygmoCor (AtCor Medical, USA) device by a single operator.

**Results:**

This study shows for the first time that isovolemic, isothermic hemodialysis neither affected blood pressure or heart rate, nor total or regional cerebral perfusion. There was also no change in pulse wave velocity.

**Conclusions:**

Mechanisms other than the dialysis procedure itself might be causative for the high incidence of ischemic strokes in this patient population. Moreover, the sole removal of uremic toxins does not lead to short-term effects on vascular stiffness, underlying the importance of volume control in this patient population.

## Introduction

The high prevalence of cardiovascular disease in chronic kidney disease (CKD) stage 5 D, i.e. dialysis patients has been known for decades [1]. Traditional cardiovascular risk markers do not solely explain these events. Functional test like the measurement of pulse wave velocity have been shown to be independent predictor of cardiovascular morbidity and mortality in this patient population [2],[3]. Despite an extensive pharmacological treatment the success in preventing or delaying cardiovascular events in CKD 5 D patients is limited [4]. Hence the dialysis procedure itself is thought to play an essential role in the pathophysiology of cardiovascular events, yet their short term impact on vascular function has not been extensively studied. For instance the effect of a single hemodialysis session on the cerebral blood flow is controversially discussed. While one group found an improvement of cerebral blood flow due to dialysis [5] other groups do see either no effect at all [6], [7] or even a decrease in cerebral blood flow [8]. Also the effect of a single hemodialysis on arterial stiffness, the result of a complex interaction between structural and functional changes in the vessel wall, has not been the focus of clinical research, making it difficult to dissect the importance of abnormal arterial calcification from volume overload uremic toxins and various endocrine abnormalities [9], [10]. Clinical studies on pulse wave velocity measurements are contradicting. Ranging from absent effects [11] to a decrease [12] or even increase [13]–[14] in pulse wave velocity (PWV) caused by dialysis, a discrepancy that might be due to either temperature effects of dialysis or the hydration status which has been shown to influence PWV [15]. Due to the scarce data about this topic, the unreliable Doppler ultrasound studies of cerebral blood flow, the aim of our study was to analyze the cerebral blood flow and the pulse wave velocity of patients with CKD 5 D before and after a single hemodialysis using the gold standard for the cerebral blood flow measurement, i.e. computer tomography based cerebral perfusion.

## Subjects and Methods

The study protocol was approved by the Hannover Medical School Ethics Committee (protocol # 3952). Stable adults undergoing chronic hemodialysis treatment were studied after written informed consent was obtained from the patients.

### Participants of the Study

Participants included were undergoing chronic hemodialysis for at least one year with a regimen of at three times per week. None of the patients suffered from instable coronary artery disease or had signs of severe heart failure (>NYHA II). The patients had no signs of infections based on c-reactive protein (CRP) and clinical examination. Exclusion criteria were participation in other studies during the previous three month, allergy against contrast agents or iodine, hyperthyroidism or unclear dysfunction of thyroid gland, premenopausal women, history of radiation of the cranium, residual diuresis (≥500 ml/day), history of stroke within six weeks prior to study participation, taking nutrition supplements containing L-arginine, and any further conditions at the discretion of the treating physician.

### Dialysis Procedure

The study participation took place on the patient’s midweek dialysis day. After measurement of the predialysis body weight, heart rate, blood pressure and body temperature the dialysis needles (17 Gauge, Bionic Medizintechnik, Friedrichsdorf, Germany) were placed as usual in the dialysis fistula and a blood gas analysis (BGA) including determining sodium and potassium was performed.

Afterwards the patient was taken to the computed tomography (CT) scan in supine and resting position, to rule out influences by altered hemodynamics. The initial CT-scan was performed as described below and the patient was taken back to the dialyis unit. The four hour lasting dialysis session was performed using the Genius® (Fresenius Medical Care, Bad Homburg Germany) single-pass batch-dialysis system with a high-flux polysulfone dialyser FX 60 (Fresenius Medical Care, Bad Homburg Germany). The dialysis solution was composed according to the patient’s requirements. The blood flow and the countercurrent dialysate flow ranged between 200 and 250 ml/min, there was no net ultrafiltration. After 4 hours the patients underwent a second pulse wave analysis and subsequently a second CT.

### Perfusion CT Imaging Protocol

The method used was described previously and adapted due to modified CT hardware [16]. In brief, all perfusion CT imaging examinations were performed at a Lightspeed 16 row helical CT scanner (GE Medical Systems, Milwaukee, Wis.). The position of the scan region was determined from a previously acquired unenhanced baseline CT. Two adjacent slices at the level of the basal ganglia included the vascular territory of the anterior, middle and posterior cerebral artery. During a scan time of 45s the total number of 90 slices for each position with a thickness of 10-mm sections of continuous (cine) scanning (80 kV, 200 mA) were obtained. CT was initiated 4s after injection (injection rate 2.5 mL/s) of iodinated contrast material with an iodine concentration of 400 mg/dl (Imeron 400, Altana, Konstanz, Germany) with a total volume of 40 mL. The contrast agent was injected via one of the placed dialysis needles followed by a saline flush with the same injection rate with a power injector (Stellant Medrad, Indianola, USA). Parametric maps of brain perfusion parameter were created from the resulting tracer kinetic images using commercially available software (Perfusion 3, AW 4.0, GE Healthcare, Milwaukee, USA). This software algorithm computed the regional cerebral blood flow (CBF) in mL/min/100 g brain tissue with the deconvolution of the parenchymal time-concentration curves by a reference arterial input function (AIF). The region of interest (ROI) that provided the AIF was placed in each anterior cerebral artery (to cover bilateral vessels); the venous outflow function ROI placed in the transverse sinus. Total cerebral perfusion was obtained by averaging blood flow from 4 standardized ROIs in each hemisphere as previously described [17].

### Measurement of Pulse Wave Velocity

Carotid-aortic PWV was determined using a validated system (Sphygmocor™; AtCor Medical, Sydney, Australia), which employs high-fidelity applanation tonometry by a pencil-type probe for non-invasive registration of peripheral arterial pressure waves. Pulse waveforms of the common carotid artery and the femoral artery were obtained sequentially and PWV was calculated as the distance between the suprasternal notch and the femoral artery recording site minus the distance between the suprasternal notch and the carotid artery recording site, divided by the time interval between the feet of the flow waves. The device uses the foot-to-foot methods as described previously [18].

### Measurement of Body Temperature

Body temperature was measured with an infrared tympanic thermometer (Genius™ 2, Covidien, Mansfield, MA, USA) with an accuracy of +/−0.1°C.

## Results

**Figure 1:**
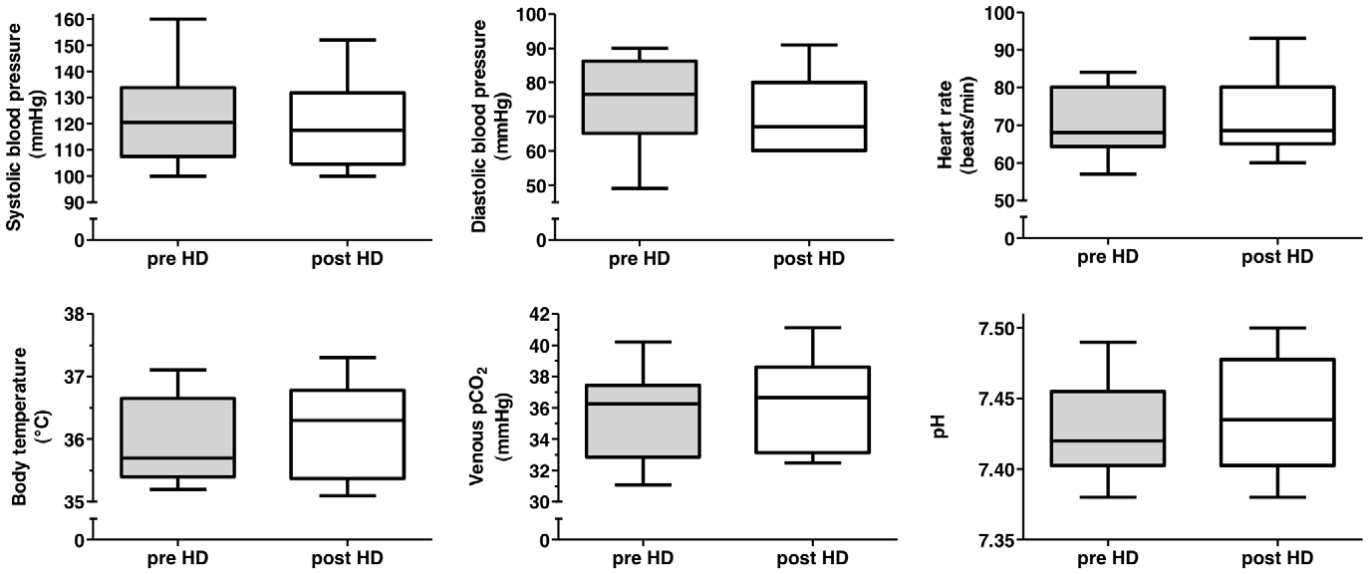
Vital signs and blood gas analysis. Predialysis and postdialysis systolic blood pressure, diastolic blood pressure, heart rate, venous pCO_2_ and pH depicted as box and whisker plots. Horizontal bars indicate median values.

**Figure 2:**
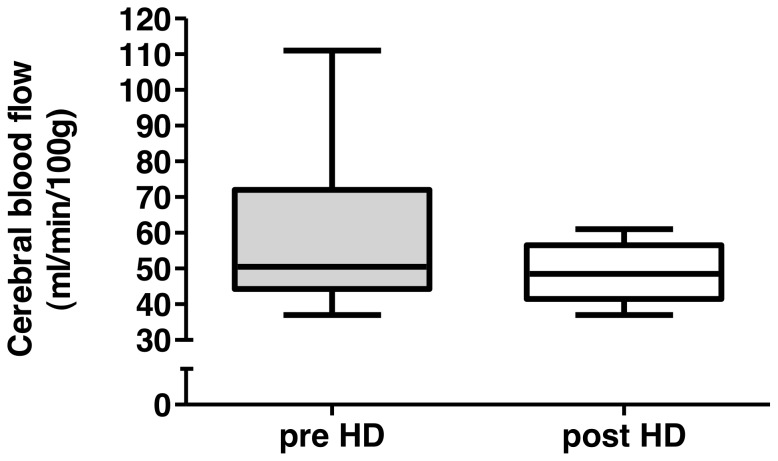
Cerebral blood flow. Predialysis and postdialysis cerebral blood flow depicted as box and whisker plots. Horizontal bars indicate median values. p = 0.16 comparing pre- and post-dialysis measurements.

**Figure 3:**
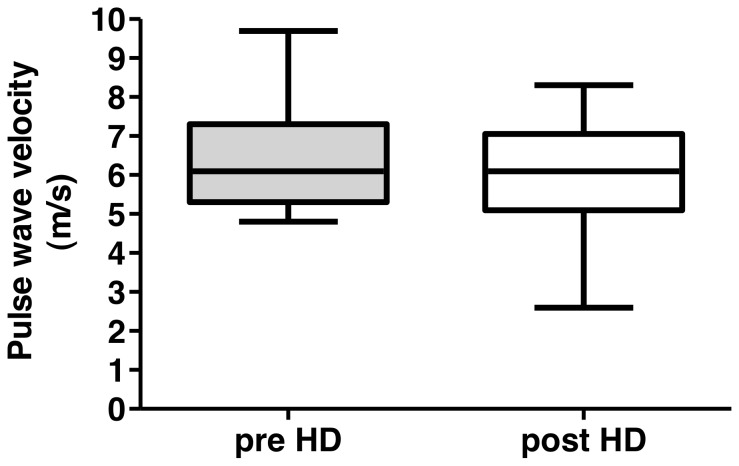
Pulse wave velocity. Predialysis and postdialysis pulse wave velocity depicted as box and whisker plots. Horizontal bars indicate median values. p = 0.39 comparing pre- and post-dialysis measurements.

Patient demographics and laboratory data are reported in **Table 1**. Isovolemic conditions could be confirmed by comparing the median [interquartile range] pre dialysis weight (71.15 [65.40–79.88] kg ) with the post dialysis weight (71.09 [65.33–79.75] kg), which revealed no difference (p = 0.9884). Isothermic hemodialysis could be confirmed by the lack of significant differences comparing pre and post dialysis body temperature (**Figure 1**). Moreover, neither systolic or diastolic blood pressure and heart rate nor pCO_2_ or pH changed significantly (**Figure 1**). There was also neither a change in total cerebral blood flow (**Figure 2**), nor in the different territories of the anterior, middle and posterior cerebral artery. Also pulse wave velocity remained unaffected by the dialysis procedure (**Figure 3**). The none-invasively measured blood pressure and heart rate, determined at 30 min intervals, did not show any significant changes over the course of the hemodialysis session (data not shown). The absolute median (range) amount of the removed uremic toxins markers in the collected spent dialysate were 428 (112–630) mmol urea and 15.4 (6.3–22.1) mmol creatinine.

**Table 1:** Patient characteristics.

Sex	Age(years)	BMI(kg/m^2^)	Dialysis(years)	Haemoglobin(g/dl)	Albumin(g/l)	AVfistula	Underlying renal disease
M	71	23.9	10.1	11.8	35	yes	nephrosclerosis
M	39	22.8	4.2	12.2	42	yes	unknown
F	55	28.8	4.5	12.0	29	yes	FSGS
M	49	22.1	4.0	11.0	30	yes	unknown
F	88	29.8	5.5	9.6	25	yes	chronic GN
M	40	23.6	2.3	9.6	41	yes	reflux nephropathy
M	57	22.1	3.5	8.9	28	yes	ADPKD
M	74	26.1	9.5	12.8	46	yes	nephrosclerosis
M	46	27.6	1.3	12.7	46	yes	membranous GN
F	69	18.4	27	12.1	43	yes	IgA nephropathy

Abbreviations: BMI = body mass index; AV-fistula = arteriovenous fistula; FSGS = focal segmental glomerulosclerosis; ADPKD = autosomal dominant polycystic kidney disease; GN = glomerulonephritis.

## Discussion

The main finding of our study is, that isovolemic, isothermic hemodialysis has neither an effect on cerebral perfusion nor in vascular elasticity in chronic hemodialysis patients.

### Pulse Wave Velocity

Pulse wave velocity is well known as an independent and highly predictive risk marker for cardiovascular events in general population [19] as well as in CKD 5D patients on hemodialysis [2], [3]. Thus far the reports on the effect of a single hemodialysis session on PWV are conflicting. Some authors found no effect of hemodialysis on PWV [11], [20] while other authors described a significant increase of PWV [13]. The latter group discussed a post-dialytic increase in heart rate and sympathetic nervous activity as causative for these findings [13]. Di Iorio et al found the marked inter- and intradialytic changes of hydration status as the major determinant for a decreasing PWV during dialysis sessions [12]. This finding is supported by our results as we did not perform a net ultrafiltration in our study, specifically to address the potential effect of volume on PWV. Indeed, in all studies investigating the effect of hemodialysis on PWV so far ultrafiltration/weight reduction ranging between ∼1500 ml [11] to ∼3.2 kg [14]. Nevertheless several of these and other authors discussed effects of the hemodialysis procedure itself, - independent of the hydration or hemodynamic effects due to ultrafiltration - like leukocyte, platelet complement activation and disordered leukocyteendothelial interactions, and increased release of oxidative radicals [11], [13], [21]. Thermal changes during dialysis strongly influence intradialytic hemodynamics [22] a truth that arose from the pioneering clinical observation of Jonas Bergstroem [23]. Indeed, beside from maintaining peripheral vascular resistance, keeping the body temperature stable also ameliorates the left ventricular dysfunction, which is often associated with hemodialysis [24]. As the sole elimination of uremic toxins in our study did not have any effect on PWV we consider volume changes and body temperature to be of major importance for short term changes in PWV.

### Cerebral Blood Flow

In our study we could not detect any effect of isovolemic, isothermic hemodialysis on cerebral blood flow, even by employing an FDA approved method, i.e. computed tomography perfusion technique [16]. Previous published studies on the effect of hemodialysis on cerebral blood flow used transcranial Doppler sonography, which is a poorly reproducible technique. The technique does not directly measure cerebral blood flow but blood flow velocity. Under conditions of constant vascular resistance cerebral blood flow can be estimated. However we know that the dialysis procedure itself may affect vascular elasticity, either decreasing [12] or increasing it [14]. In contrast to all those studies we could not see a significant change of the cerebral blood flow while eliminating uremic toxins. As we kept blood pressure and the heart rate constant, we could exclude that volume alterations interfered the cerebral blood flow measurements. This could explain the difference to other studies [5], [8] in which a marked rate of ultrafiltration of about 2200 ml was performed. Beside ultrafiltration and the loss of body weight the hemoconcentration was associated with change of cerebral flow in this studies. In our study with no net ultrafiltration those parameters were kept stable. Therefore we postulate that the ultrafiltration during dialysis plays a crucial role for the decreased blood flow seen by other authors. Furthermore we conclude that the solute removal itself has no influence on cerebral blood flow. This assumption is supported by data showing that high interdialytic weight gain is associated with a high mortality [25]. Higher rates of ultrafiltration are associated with significant increase in all-cause and cardiovascular mortality, probably induced by repetitive transient cardiac ischemia due to the intravascular hypovolemia [26]. The same effect might happen to the brain that suffers from cyclic states of reduced perfusion following ultrafiltration during hemodialysis. Accelerated aging of the brain due to reduced cerebral blood flow has also been proposed as an important mechanism in the Framingham population [27].

### Limitations of the Study

We wish to point out important limitations of our study. Firstly the number of subjects studied is rather small, yet the use of state of the art methodology subjecting patients to an x-ray based procedure. Secondly, we did not perform a net ultrafiltration, as we felt that had been done in several studies performed previously. Thirdly due to methodology it was not possible to measure cerebral blood flow during the dialysis session, therefore we cannot rule out intermediate fluctuations. Fourthly, patients were treated with the GENIUS dialysis system which has some peculiar properties, such as the absence of an in-built heating. Nevertheless, a drop in body temperature had so far not been reported over the course of a four hour treatment [28], [29]. Last but not least we specifically excluded patients with diabetic nephropathy.

In summary, during a single hemodialysis session, the removal of uremic toxins alone had neither an effect on vascular stiffness nor on cerebral blood flow. Hence most like the combination of long term changes in the vessel wall anatomy in combination with short term volume shifts might be responsible for the high prevalence of asymptomatic silent cerebral infarction in CKD 5 D patients.

## Supporting Information

Figure S1 (TIFF)
