# Correction: Effect of Isovolemic, Isothermic Hemodialysis on Cerebral Perfusion and Vascular Stiffness Using Contrast Computed Tomography and Pulse Wave Velocity

**DOI:** 10.1371/annotation/b8510ff9-3ea4-4415-bc1b-5fa7b341f293

**Published:** 2013-05-02

**Authors:** Ansgar Reising, Saskia Sambale, Frank Donnerstag, Julius J. Schmidt, Carsten Hafer, Bernhard M.W. Schmidt, Jan T. Kielstein

There was an error in the Funding statement. The correct version of the Funding is:

This study was supported by an unrestricted grant of the Else-Kröner-Fresenius Foundation (P63/06//EKMS 06/03). The publication of the study is supported by the DFG-project "Open Access Publication" at the Hannover Medical School. The funders had no role in study design, data collection and analysis, decision to publish, or preparation of the manuscript. 

