# Supplementary figures and images for: Effect of Isovolemic, Isothermic Hemodialysis on Cerebral Perfusion and Vascular Stiffness Using Contrast Computed Tomography and Pulse Wave Velocity
Source: PLoS One. 2013 Feb 22;8(2):e56396. doi: 10.1371/journal.pone.0056396 (PMC3579888; doi:10.1371/journal.pone.0056396)

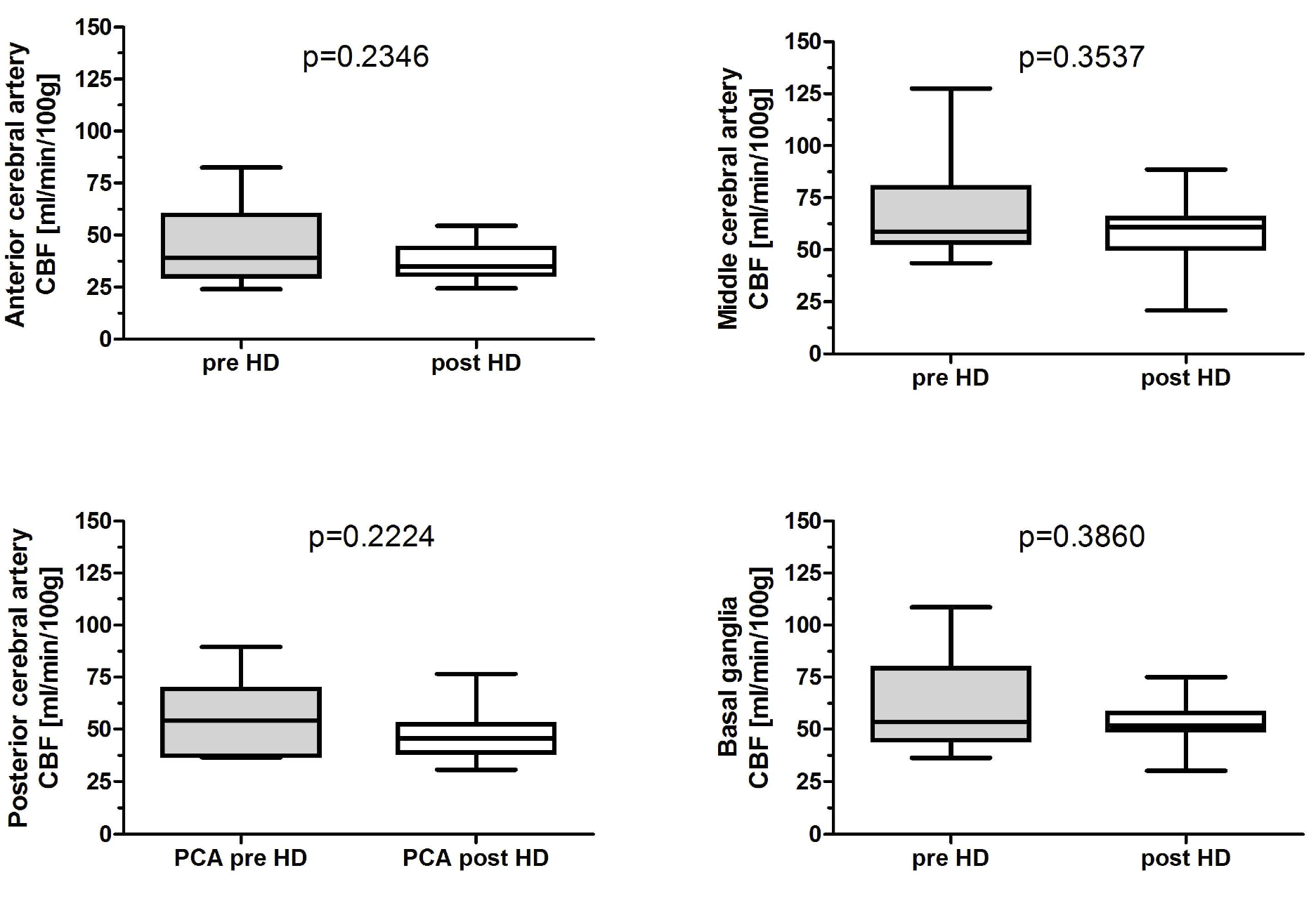

Supplement: Figure S1 — (TIFF) [file pone.0056396.s001.tif]
